# Supplementary material for: Is there a placental microbiota? A critical review and re-analysis of published placental microbiota datasets
Source: BMC Microbiol. 2023 Mar 18;23:76. doi: 10.1186/s12866-023-02764-6 (PMC10024458; doi:10.1186/s12866-023-02764-6)
Supplement: Supplementary file 1 — Additional file 1. [file 12866_2023_2764_MOESM1_ESM.pdf]

Figure S1. World map of study locations

World map indicating the locations of studies included in this re-analysis.

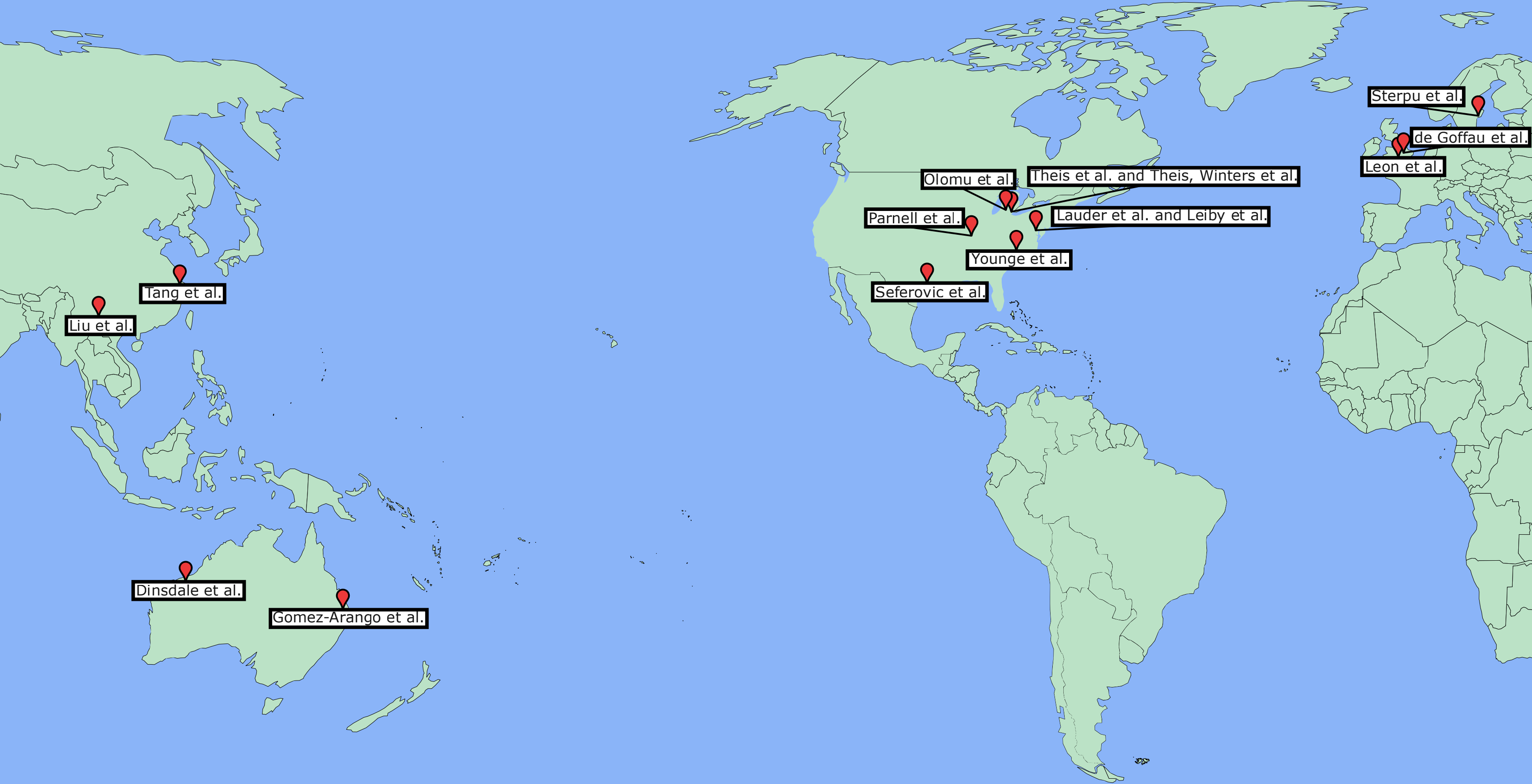

Tang et al.
Liu et al.
Dinsdale et al.
Gomez-Arango et al.
Olomu et al.
Theis et al. and Theis, Winters et al.
Parnell et al.
Lauder et al. and Leiby et al.
Younge et al.
Seferovic et al.
Sterpu et al.
de Goffau et al.
Leon et al.

Table S1. Summary of the genus level classifications of prominent bacterial ASVs across studies unable to be subset to placentas from term cesarean deliveries before and after use of the R package DECONTAM to remove likely background DNA contaminants

Top five ASV genus level classifications as determined by mean relative abundance across placental samples after DADA2 processing for studies which had ASVs greater than 1% mean relative abundance across all placental samples. The de Goffau et al. and Tang et al. studies were omitted from the table because there were no ASVs which contributed on average more than 1% to placental sample reads; however, reads from *Cutibacterium* and *Romboutsia* were most predominant in the de Goffau and Tang datasets, respectively. The 16S rRNA gene hypervariable regions sequenced are included, as well as why placental samples in a study could not be subset to only cesarean term delivered samples. Asterisks indicate ASV sequence genus level classifications which were assigned to type strains by NCBI BLAST with the highest percent identity in excess of 95%.

Study	16S rRNA gene hypervariable region	Before DECONTAM				
		ASV1	ASV2	ASV3	ASV4	ASV5
		**Fetal-side** [a]				
Olomu	V4	*Escherichia/ Shigella*	***Lactobacillus***	***Lactobacillus***	*Finegoldia*	*Fenollaria*
Theis	V4	*Achromobacter*	*Delftia*	*Phyllobacterium*	*Clostridium sensu stricto 5*	*Stenotrophomonas*
Theis, Winters	V4	*Escherichia/ Shigella*	*Staphylococcus*	*Serratia**	*Cupriavidus*	*Corynebacterium*
Sterpu	V6-V8	*Cutibacterium*	*Staphylococcus*	*Streptococcus*	*Staphylococcus*	*Aggregatibacter*
		**Maternal-side** [b]				
Theis, Winters	V4	*Escherichia/ Shigella*	***Lactobacillus***	*Staphylococcus*	*Serratia**	*Streptococcus*
Sterpu	V6-V8	*Cutibacterium*	*Streptococcus*	*Staphylococcus*	*Streptococcus*	*Streptococcus*
		**After DECONTAM**				
		ASV1	ASV2	ASV3	ASV4	ASV5
		**Fetal-side** [a]				
Olomu	V4	*Fenollaria*	*Acinetobacter*	***Lactobacillus***	*Campylobacter*	*Peptoniphilus*
Theis	V4	*Achromobacter*	*Alcaligenaceae*	*Escherichia/ Shigella*	*Achromobacter*	*Alcaligenaceae*
Theis, Winters	V4	*Serratia**	*Corynebacterium*	*Cupriavidus*	*Corynebacterium*	*Achromobacter*
Sterpu	V6-V8	*Staphylococcus*	*Staphylococcus*	*Cutibacterium*	*Staphylococcus*	
		**Maternal-side** [b]				
Theis, Winters	V4	*Corynebacterium*	***Lactobacillus***	*Serratia**	*Peptoniphilus*	*Corynebacterium*
Sterpu	V6-V8	*Staphylococcus*	*Staphylococcus*	*Cutibacterium*	*Staphylococcus*	

[a] Samples labeled 'amnion', 'amnion-chorion', 'amnion-chorion interface', 'sub-chorionic plate swab', 'placental parenchyma', 'villous tree' or 'fetal' were categorized as placental samples from the fetal side.

[b] Samples labeled 'decidua' or 'basal plate' were categorized as placental samples from the maternal side.

ASV1-5 are rank designations based on percent relative abundance.

Lactobacillus ASVs are bolded to emphasize prevalence and lack thereof after likely-contaminant removal.

Table S2. Summary of the genus level classifications of prominent bacterial ASVs across studies which sampled placental tissues of fetal and maternal origin from term cesarean deliveries without labor

Results are presented before and after the use of the R package DECONTAM to remove likely background DNA contaminants. The top five ASV genus level classifications as determined by mean relative abundance across placental samples after DADA2 processing are provided for each study. Placental samples are separated based on fetal or maternal origin. Asterisks indicate ASV sequence genus level classifications which were assigned to type strains by NCBI BLAST with the highest percent identity in excess of 95%.

Study	Level	N (placental samples - controls)	Dispersion		NPMANOVA		
			F Statistic	P value	R^2	F Statistic	P Value
de Goffau	Villous tissue	77 - 45	6590	**$1x10^{-106}$**	0.00825	0.999	0.636
Parnell	Fetal membrane	54 - 11	0.0885	0.767	0.0159	1.02	0.339
	Placental villus	54 - 11	0.0488	0.826	0.0149	0.952	0.432
	Basal plate	55 - 11	0.00526	0.942	0.0186	1.21	0.169
Olomu	Placental parenchyma	23 - 24	0.698	0.408	0.016	0.731	0.75
Sterpu	Fetal	46 - 6	12.1	**0.00105**	0.0736	3.97	**0.001**[a]
	Middle	46 - 6	4.64	**0.0361**	0.0761	4.12	**0.001**[a]
	Decidua	43 - 6	9.46	**0.0035**	0.0749	3.81	**0.001**[a]
Theis	Amnion-chorion	28 - 42	2.04	0.158	0.0205	1.42	0.141
	Villous tree	29 - 42	2.02	0.16	0.0285	2.03	**0.033**[b]
Theis, Winters	Amnion	129 - 12	3.67	0.0617	0.0862	4.43	**0.001**[c]
	Amnion-chorion	97- 12	0.23	0.635	0.0371	1.23	0.128
	Subchorion	61 - 12	1.12	0.302	0.053	1.23	0.193
	Villous tree	48 - 12	0.572	0.46	0.0661	1.13	0.258
	Basal plate	114 - 12	0.00843	0.927	0.035	1.42	**0.041**[c]

[a] ADONIS significance is likely to be due to differences in dispersion and unbalanced groups. Regardless, *Staphylococcus* was predominant in samples responsible for significance.

[b] The bacterial profiles of room samples (exposed to room air for 20 minutes), which were predominantly characterized by *Delftia*, *Blastomonas*, and *Achromobacter* from the Theis et al. dataset were responsible for ADONIS significance, which disappeared after their removal from the analysis.

[c] Amnion and basal plate samples driving significance were characterized by *Staphylococcus*, *Corynebacterium*, *Serratia*, and *Cupriavidus*.
